# Moderation of the Association Between Chronic Medical Conditions and Functional Limitations Over Time by Physical Activity: Effects of Age

**DOI:** 10.1093/gerona/glz020

**Published:** 2019-01-21

**Authors:** Jerrald L Rector, Kristine Marceau, Elliot M Friedman

**Affiliations:** Human Development and Family Studies, Purdue University, West Lafayette, Indiana

**Keywords:** Chronic medical conditions, Disability, Functional limitations, Physical activity

## Abstract

**Background:**

Age-related accumulation of chronic medical conditions increases disability in older adults. Physical activity potently combats chronic conditions and disability. However, it is unclear whether activity maintenance alleviates the effects of chronic conditions on disability and if this buffering effect differs with age. This study examined whether long-term physical activity can forestall functional limitations in the face of accumulating chronic conditions among middle-aged and older adults.

**Methods:**

Participants (*n* = 2,119; 54.7% female) were from the Survey of Midlife Development in the United States. Self-reported physical activity, number of chronic conditions, and functional limitations were obtained across 18–20 years. Functional limitations were regressed against the change in chronic conditions, physical activity, and their interaction over time in a multilevel model of change. Baseline age was added as an additional moderator.

**Results:**

Faster accumulation of chronic conditions [B(*SE*) = 2.08(0.32), *p* < .001] and steeper declines in activity [B(*SE*) = −2.29(0.41), *p* < .001] were associated with greater increases in functional limitations over time. Among those with faster-than-average increases in conditions, those who maintained activity had a slower progression of functional limitations, compared to those whose activity declined more rapidly [B(*SE*) = −11.18(3.96), *p* = .005]. Baseline age moderated the buffering effect of activity maintenance; older adults were protected against functional limitations only when conditions accumulated slowly [B(*SE*) = 0.23(0.08), *p* = .005].

**Conclusion:**

This study provides evidence for an age-dependent buffering effect of activity maintenance on the longitudinal relationship between chronic conditions and functional limitations. Intervention strategies using physical activity to forestall disability should target midlife adults and consider the rate of condition accumulation.

An estimated 10.6% of working-aged (18–64 years old) and 35.2% of older (≥65 years) adults have at least one disability, with mobility-related disabilities being most prevalent (1–3), and the prevalence of disability is expected to increase substantially with the expanding proportion of older adults in the population (4). Functional capacity (e.g., strength, mobility) often declines before overt disability (5). The determinants of functional impairments are diverse, but the ways in which they interact to predict change in functional capacity over time are rarely examined. This study focuses on functional limitations as a potential precursor to disability according to The Disablement Process Model (5), and specifically considers the interactive effects of two factors with opposing influences on functional limitations over time: chronic medical conditions and physical activity.

Age-related accumulation of chronic medical conditions (e.g., arthritis, cardiovascular disease) is known to contribute to increased disability (6,7). Further, the co-occurrence of multiple chronic conditions, or multimorbidity, is associated with a greater likelihood of disability beyond that attributable to the individual diseases (8), suggesting synergistic effects. Although most studies focus on older adults, a considerable proportion of individuals with multimorbidity are working-aged (9). Consistent with a life-span perspective on health (10), illness and functional decline in midlife could result in disability in later life. This study takes advantage of data from middle-aged and older adults to investigate the long-term impact of accumulating chronic conditions on functional limitations across adulthood.

Regular physical activity participation has a wide range of beneficial effects on health, including improving physical functioning and reducing disability (11). Longitudinal studies generally show that higher levels of activity are protective in older adults, either by preventing disability onset or by slowing its progression among those with preexisting disability (12,13). Studies involving adults at least 50 years old show that those more physically active have a significantly reduced risk of incident functional limitations and disability compared to those less active (14,15).

Importantly, a prospective study of adults aged 25–74 years from the National Health and Nutrition Examination Survey showed that greater baseline physical activity and increases in physical activity over time were associated with reduced disability risk 10 years later (16). Levels of, and changes in, activity over time can thus have long-term effects on disability, which are discernible in middle-aged and older individuals. However, that study was unable to simultaneously model change in activity and disability over the entire 20-year follow-up period. This study addresses this gap by examining how long-term physical activity and functional limitations covary over time.

The interactive influence of physical activity and chronic conditions on the trajectory of functional limitations is unclear. Studies across diverse populations have documented largely consistent inverse relationships between physical activity levels and multimorbidity at a given time point (17,18). Although cross-sectional evidence suggests that increased numbers of chronic conditions may be linked to lower levels of physical activity (19–21), increased physical activity likely lowers the prevalence of multimorbidity via concomitant increases in muscle strength and/or cardiorespiratory fitness (22). This study explicitly examines the interactive influence of these factors on functional limitations as they both change over time.

Finally, the extent to which physical activity mitigates the adverse effects of accumulating chronic conditions on disability may vary with age. Although it is clear that activity maintenance in older adults may improve muscular strength and aerobic capacity, it is less clear whether late-life activity can minimize or prevent functional decline and disability (23,24). This study examines age differences in the extent to which long-term physical activity participation protects against functional decline in the face of accumulating chronic conditions.

This study extends the literature in three ways. First, we examine long-term trajectories of chronic conditions, physical activity, and functional limitations in a large, age-diverse, national sample over a 20-year follow-up period. We predict that increases in multimorbidity and declines in physical activity will be associated with greater increases in functional limitations over time. Second, we test the central hypothesis that physical activity may be protective against functional declines in adults with multimorbidity by examining the interaction between multimorbidity and physical activity over time. Finally, to determine whether the potential buffering effect of physical activity on the trajectory of functional limitations is age-dependent, we include baseline age as an additional moderator of the conditions–limitations relationship.

## Method

### Participants

Participants were from the longitudinal Midlife in the United States (MIDUS) study. MIDUS comprises a national probability sample of noninstitutionalized English-speaking adults living in the co-terminus United States. The first wave of data (MIDUS 1; *n* = 7,108) was collected from 1995 to 1996. In addition to a random digit dialing sample, data were also included from siblings of random digit dialing participants, oversamples from five metropolitan areas, and a national registry of twin pairs. Follow-up assessments were completed from 2004 to 2006 (MIDUS 2; *n* = 4,032) and 2013–2014 (MIDUS 3; *n* = 3,294). Mortality-adjusted retention rates were 75% and 77% at MIDUS 2 and MIDUS 3, respectively.

Participants at all three MIDUS waves completed telephone interviews and self-administered questionnaires; only participants who completed both were included in this study. Of the 6,325 participants with available data at MIDUS 1, data on functional limitations from all three waves were available for 2,490 respondents. Participants were excluded if they had missing data on physical activity (*n* = 147), chronic conditions (*n* = 1), sociodemographics (*n* = 27), lifestyle factors (*n* = 195), or self-rated health (*n* = 1) at any wave, leaving 2,119 participants with full information for all three waves. Compared to those excluded (*n* = 371, 14.9%), the final analytic sample was significantly younger (45.9 vs 51.0 years; *p* < .001), more likely to be male (45.3% vs 36.7%; *p* = .002) and to have higher education (42.3% vs 29.4% with a 4-year degree or more; *p* < .001). Including only those with information at all waves necessarily introduces selection bias, which the reader should take into account.

### Functional Limitations

Participants were asked how much their health limited their ability to carry out any of seven activities, including “lifting or carrying groceries,” “bathing or dressing yourself,” “climbing stairs,” “bending, kneeling, or stooping,” and walking “one block”, “several blocks”, or “more than a mile” (1 = “Not at all”; 4 = “A lot”). These items assessed the extent of health-related functional limitations to basic and instrumental activities of daily living. For participants with at least five of seven non-missing responses, these items were averaged yielding a continuous score from 1 to 4.

### Chronic Conditions

The number of chronic medical conditions was obtained from participant self-report. Heart problems and cancer were assessed via telephone interview by the questions “Have you ever had heart trouble suspected or confirmed by a doctor?” and “Have you ever had cancer?” Information on hypertension, asthma, arthritis, diabetes, autoimmune disorders, neurological disorders, stroke, migraine, and depression was obtained from the self-administered questionnaire where participants were asked “In the past 12 months, have you experienced or been treated for any of the following?” Information on high cholesterol was derived from medication use. Participants with a body mass index at least 30, calculated from self-reported height and weight, were categorized as obese. “Yes” responses were then summed to create a continuous chronic conditions score (range: 0–13). As there is no generally accepted definition of multimorbidity, selection of these specific conditions was informed by multiple clinical, epidemiological, and public health sources. For example, the conditions included here are the most common ailments of middle-aged and older adults and appear in different formulations of multimorbidity. They are also among the most clinically severe and most likely to result in adverse health outcomes according to the Charlson Comorbidity Index (25).

### Physical Activity

Participants reported their frequency of moderate (“bowling or using a vacuum cleaner”) and vigorous (“running or lifting heavy objects long enough to work up a sweat”) physical activity during the summer and winter from 0 = “Never” to 5 = “Several times a week or more.” Responses (2 intensities × 2 seasons) were used as indicators to estimate a continuous latent physical activity variable at each wave. When compared to population estimates from the Center for Disease Control and Prevention (CDC), the proportions of individuals within activity classifications (inactive, not regularly active, and regularly active) derived from these MIDUS measures were found to be similar but may overestimate physical inactivity due to reports being from summer and winter only (26).

### Covariates

A dichotomous variable for sex (1 = female) and a continuous variable for participant age were included in the analyses. Baseline age was also considered as a predictor of interest (see later). Educational attainment was determined from a telephone interview question, and a categorical variable (1 = “high school diploma or equivalent,” 2 = “some college,” 3 = “4-year college degree or more”) was included in all models. Marital status was included as a dichotomous variable (1 = Married). Given the small number of racial minoritiy respondents in the overall sample (~6%), a dichotomous variable for race (1 = “Minoritiy”) was used. Lifestyle factors included continuous body mass index and categorical smoking status (1 = “nonsmoker,” 2 = “ex-smoker,” 3 = “current smoker”) determined from participant-reported height and weight and smoking behavior, respectively. Finally, self-rated health was assessed by asking participants to rate their general physical health from 1 = “poor” to 5 = “excellent.” With the exception of baseline age, sex, and race, all covariates were included as time-varying factors in the analyses.

### Statistical Analyses

Analyses were conducted in two steps: data preparation and subsequent hypothesis testing. Because the primary interest was testing whether between-person differences in the slope of change in chronic conditions and physical activity over time were associated with functional limitations, an initial “de-trending” process was first applied to extract a meaningful measure of between-person change over time in the predictors that was not confounded by their within-person variability at individual time points (27). Specifically, de-trending entails regressing the repeated measures of each predictor on time, coded in terms of years in the study (centered so that 0 indicates the initial assessment, MIDUS 1), in separate multilevel models. The Bayes empirical estimates for the intercept and slope, representing the starting point and change over time for each predictor, respectively (i.e., measures of between-person differences), and residuals, representing deviations in individuals’ actual scores from the model estimate (i.e., a time-varying measure of within-person variability), for chronic conditions and physical activity were extracted for use in hypothesis testing. Slopes and intercepts were grand mean-centered; residuals were within-person centered.

The second, hypothesis-testing step comprised a multilevel model of change with the repeated measures of functional limitations as the dependent variable. As the central aim of this study was to examine longitudinal associations, the effects of slopes (indicating change over time) of chronic conditions and physical activity and their interaction on functional limitations were of primary interest. Model building occurred in three steps. The first model included the slope of change in physical activity as a moderator of the relationship between the slope of change in chronic conditions and that of functional limitations over time (along with the main effects of time, change in chronic conditions and physical activity, and all lower-order interactions). These analyses were adjusted for potential confounders, including the time-varying effects of age, education, marital status, body mass index, smoking status, and self-rated physical health (all within-person centered), and the between-person effects of sex and race.

In the second model, the intercepts and residuals of chronic conditions and physical activity from the de-trending step were added as covariates to additionally adjust for the initial levels of (between-person differences) and time-specific variation in (within-person effects) conditions and activity on functional limitations, respectively. These variables were intentionally added in a separate model to highlight the predictor-related effects that are typically confounded without de-trending. Finally, a third model was fit to investigate whether the ability of long-term physical activity participation to buffer the relationship between the change in chronic conditions and limitations over time is age-dependent (23). In this final model, the four-way interaction between change in chronic conditions, change in physical activity, time, and baseline age was estimated, including all lower-order interactions and main effects. The same covariates were included as in Model 2, although age was no longer included as a time-varying covariate. Owing to skew in the outcome variable, linear mixed models with a Huber–White sandwich variance estimator was used for all analyses to improve robustness to violations of the model assumptions (28). All analyses were conducted using Stata version 14 (Stata Corp., College Station, TX).

## Results

Table 1 shows the participant characteristics at each wave of MIDUS. De-trending analyses revealed that the average number of chronic conditions was 1.06 and participants engaged in moderate-to-vigorous activity several times per month (score of 3.39). There was an overall positive slope for chronic conditions, (*M* = 0.06), and a negative slope for physical activity (*M* = −0.06; both *p* < .001). To contextualize these changes, an individual 1 *SD* above average would add 1 condition per decade (steeper increase), compared to approximately 0.2 conditions for those 1 *SD* below average (gradual increase). An individual with changes in activity 1 *SD* above average (flatter slopes) would maintain engagement in physical activity at approximately 2–3 times a month across 10 years, whereas changes 1 *SD* below average (faster declines) would be equivalent to an individual dropping to once a month or less over the same period.

**Table 1.** Participant Characteristics (*N* = 2,119)

	MIDUS 1	MIDUS 2	MIDUS 3
Time since baseline, years	—	8.90 (0.64)	17.99 (0.65)
Age, years	45.89 (11.00)	54.79 (10.93)	63.88 (10.95)
Sex (female), %	54.7	—	—
Race (White), %	94.5	—	—
Marital status (married), %	34.3	34.2	31.5
Education, %			
High school/GED or less	34.6	33.0	32.4
Some college	34.4	32.5	33.1
College or more	31.9	34.1	34.0
Body mass index, kg/m^2^	26.46 (5.07)	27.70 (5.53)	28.11 (5.97)
Smoking status, %			
Never smoker	34.1	33.1	32.8
Ex-smoker	28.7	33.3	38.0
Current smoker	43.3	34.2	22.5
Self-rated health (1–5)*	3.74 (0.89)	3.75 (0.91)	3.51 (1.00)
Chronic conditions (0–13)^*,†^	1.00 (2.00)	1.00 (2.00)	2.00 (2.00)
Physical activity (0–5)*	3.53 (0.75)	2.60 (1.24)	2.51 (1.25)
Functional limitations (1–4)^*, †^	1.00 (0.29)	1.14 (0.57)	1.29 (1.14)

*Notes:* Values are mean (*SD*) unless otherwise specified. MIDUS = Midlife in the United States; GED = General Education Diploma.

*Higher values indicate higher self-rated health, greater number of chronic conditions, more frequent physical activity, and greater functional limitations.

^†^Values are median (IQR).

**Figure 1.**
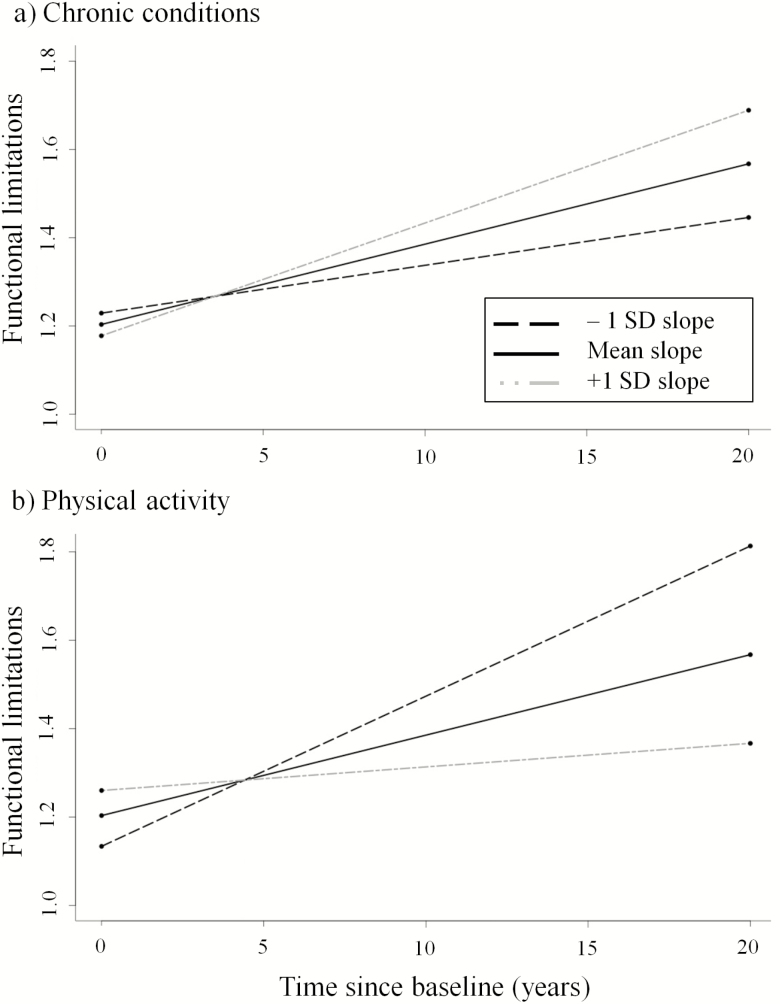
Functional limitations over time as a function of the longitudinal slopes (mean and ±1 *SD*) of (**a**) chronic medical conditions and (**b**) physical activity.

Table 2 shows the effects of chronic conditions and physical activity on functional limitations. Results from the first model showed that after adjustment for potential confounders, increases in chronic conditions [B(*SE*) = 2.08(0.32), *p* < .001] and declines in physical activity [B(*SE*) = −2.29(0.41), *p* < .001] were associated with greater limitations at a given time point. Of particular interest, the change in conditions and in physical activity were both independently associated with the slope of limitations over time (Conditions × Time: B(*SE*) = 0.21(0.02), *p* < .001; Activity × Time: B(*SE*) = −0.32(0.03), *p* < .001). For illustrative purposes, these two interactions are shown in Figure 1. Specifically, increases in the numbers of chronic conditions and decreases in physical activity over time predicted greater increases in limitations over time, consistent with the first hypothesis. However, the three-way interaction between conditions, activity, and time was nonsignificant (see Model 1, Table 2), suggesting sustained activity participation did not buffer functional declines among participants with increasing numbers of conditions.

**Table 2.** Effects of Chronic Conditions and Physical Activity on Functional Limitations (*N* = 2,119)

	Model 1			Model 2			Model 3		
	Coefficient	*SE*	*p* value	Coefficient	*SE*	*p* value	Coefficient	*SE*	*p* value
Fixed-effects									
Intercept	1.19	0.01	**<.001**	1.21	0.01	**<.001**	1.22	0.05	**<.001**
Time, years	0.02	0.00	**<.001**	0.02	0.00	**<.001**	−0.01	0.00	**.004**
Slopes									
Conditions	2.08	0.32	**<.001**	−0.37	0.33	.253	−0.26	1.22	.834
Activity	−2.29	0.41	**<.001**	1.26	0.59	**.032**	1.28	1.62	.429
Conditions × Time	0.21	0.02	**<.001**	0.20	0.02	**<.001**	0.11	0.11	.281
Activity × Time	−0.32	0.03	**<.001**	−0.29	0.03	**<.001**	−0.12	0.14	.365
Conditions × Activity	−16.07	11.88	.176	−13.71	10.34	.185	−47.61	47.72	.318
Conditions × Activity × Time	−0.10	0.83	.905	−0.08	0.83	.922	−11.18	3.96	**.005**
Conditions × M1 age	—	—	—	—	—	—	0.00	0.03	.908
Activity × M1 age	—	—	—	—	—	—	−0.01	0.03	.843
Time × M1 age	—	—	—	—	—	—	0.00	0.00	**<.001**
Conditions × Time × M1 age	—	—	—	—	—	—	0.00	0.00	.539
Activity × Time × M1 age	—	—	—	—	—	—	0.00	0.00	.315
Conditions × Activity × M1 age	—	—	—	—	—	—	0.74	0.95	.434
Conditions × Activity × time × M1 age	—	—	—	—	—	—	0.23	0.08	**.005**
Intercept									
Conditions	—	—	—	0.26	0.02	**<.001**	0.26	0.02	**<.001**
Activity	—	—	—	−0.33	0.06	**<.001**	−0.33	0.06	**<.001**
Residual									
Conditions	—	—	—	0.02	0.01	.173	0.02	0.01	.167
Activity	—	—	—	−0.02	0.01	**.005**	−0.02	0.01	**.006**
Covariates									
Age (person-centered)	0.01	0.00	**<.001**	0.00	0.00	**<.001**	—	—	—
M1 age (grand mean-centered)	—	—	—	—	—	—	0.00	0.00	0.971
Sex (female = 1)	0.16	0.02	**<.001**	0.12	0.02	**<.001**	0.12	0.02	**<.001**
Race (Minority = 1)	0.02	0.05	.702	−0.03	0.05	.567	−0.02	0.05	.594
Education (person-centered)	−0.01	0.02	.501	−0.02	0.02	.433	−0.01	0.02	.748
Marital status (person-centered)	−0.07	0.02	**.001**	−0.07	0.02	**.001**	−0.05	0.02	**.018**
Body mass index (person-centered)	0.02	0.00	**<.001**	0.01	0.00	**<.001**	0.02	0.00	**<.001**
Smoking status (person-centered)	0.00	0.03	.898	−0.01	0.03	.777	−0.01	0.03	.647
Self-rated health (person-centered)	−0.15	0.01	**<.001**	−0.14	0.01	**<.001**	−0.14	0.01	**<.001**
	Model 1			Model 2			Model 3		
	Estimate	SE	(95% CI)	Estimate	SE	(95% CI)	Estimate	SE	(95% CI)
Random-effects									
Time	0.00	0.00	(0.00–0.00)	0.00	0.00	(0.00–0.00)	0.00	0.00	(0.00–0.00)
Intercept	0.13	0.01	(0.11–0.16)	0.09	0.01	(0.07–0.10)	0.09	0.01	(0.07–0.10)
Residual	0.13	0.01	(0.12–0.15)	0.14	0.01	(0.12–0.15)	0.13	0.01	(0.12–0.15)

*Notes:* Significant *p* values are bolded. CI = Confidence interval; M1 age = age at Midlife in the United States (MIDUS) baseline.

The above interactions with time (i.e., Conditions × Time and Activity × Time) remained after additional adjustment for the intercepts and residuals of the predictors (see Slope, Model 2, Table 2), suggesting the changes in conditions and activity are associated with changes in functional limitations independent of participants’ initial levels and time-specific deviations.

**Figure 2.**
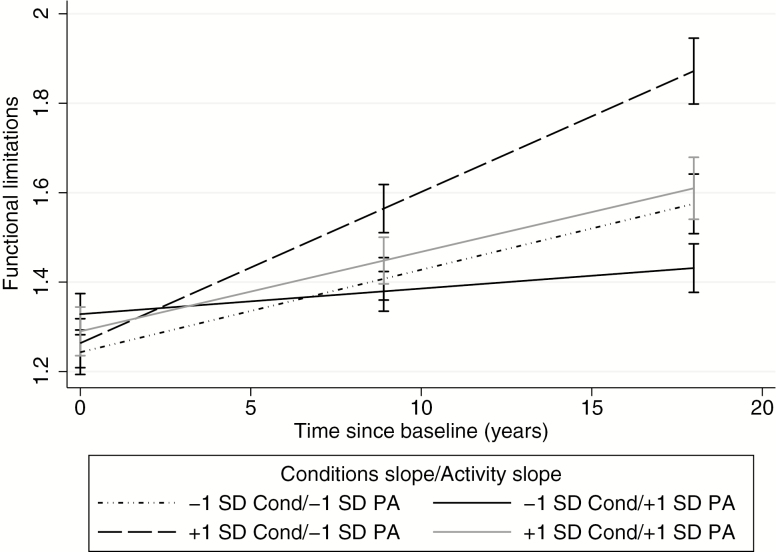
Change in functional limitations over time as a function of change in chronic conditions and the rate of decline in physical activity (mean slope ± 1 *SD*). Cond = chronic conditions slope; PA = physical activity slope. Error bars are 95% confidence intervals.

Finally, to examine the buffering capacity of long-term physical activity as a function of age, a third model was fit including baseline age as an additional moderator. The three-way interaction between the conditions slope, activity slope, and time was significant, B(*SE*) = −11.18(3.96), *p* = .005 (see Slope, Model 3, Table 2). Visualizing this interaction, Figure 2 shows that those with steeper increases in chronic conditions (slopes 1 *SD* above average) who also had faster declines in physical activity showed the most rapid increase in limitations over time. Conversely, those with more gradual increases in chronic conditions (slopes 1 *SD* below average) and flatter physical activity slopes—indicative of long-term activity maintenance—had the slowest rise in limitations over time. Consistent with the central hypothesis, among those with steeper increases in conditions, those that simultaneously maintained physical activity had significantly lower limitations at MIDUS 2 and MIDUS 3 than those whose physical activity declined more rapidly (solid gray line vs long-dashed line in Figure 2).

**Figure 3.**
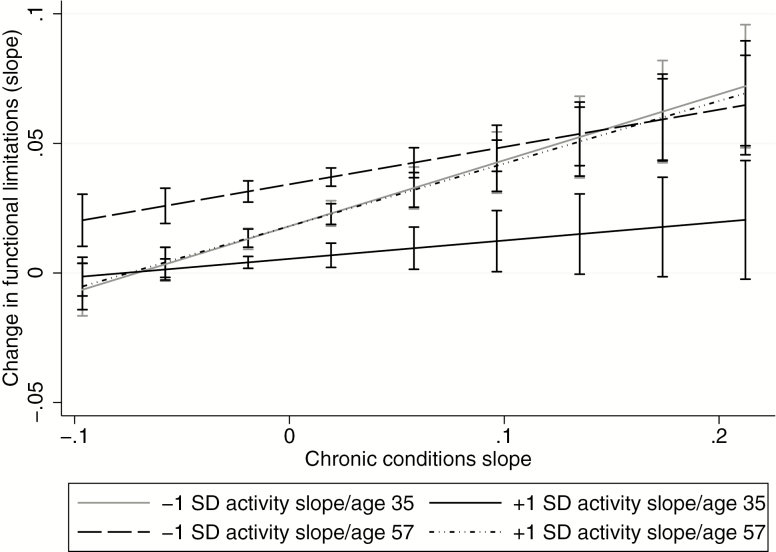
Relationship between change in chronic conditions (mean-centered) and change in functional limitations as a function of rate of decline in physical activity (mean slope ± 1 *SD*) and baseline age (average age ± 1 *SD*). The values on y-axis represent slope of change in functional limitations over time. The values on x-axis represent rate of change in chronic conditions over time. Age 35 and age 57 represent 1 *SD* below and above the average baseline age, respectively. Error bars are 95% confidence intervals.

Interestingly, the four-way interaction was also significant, B(*SE*) = 0.23(0.08), *p* = .005 (see Slope, Model 3, Table 2). Figure 3 shows the age-dependent moderating effect of long-term physical activity participation on the association between conditions and limitations over time. There were three notable patterns. First, younger participants who maintained activity showed little increase in limitations over time (i.e., near-zero slope) regardless of change in chronic conditions (solid black line). Second, older adults with more rapid declines in activity had significant increases in limitations that increased in magnitude with steeper increases in chronic conditions over time (higher values on the x-axis; long-dashed line in Figure 3). Finally, young adults with more rapidly declining activity and older adults with sustained activity showed a similar pattern: limitations did not increase over time when there were only gradual increases in chronic conditions, but change (i.e., slope) in limitations increased significantly and was of greater magnitude when conditions accumulated faster over time (solid gray and dotted-dashed line).

Overall, activity maintenance buffered the relationship between conditions and limitations over time across the range of chronic condition slopes among younger individuals, and among older individuals with slower progressions of conditions.

## Discussion

This study examined the interactive influences among trajectories of physical activity engagement, chronic medical conditions and functional limitations over 20 years among middle-aged and older adults. The results suggest that the trajectories of both chronic conditions and physical activity independently affected change in limitations over time, after controlling for potential confounders. Of note, the final model, including baseline age as an additional moderator, revealed that the buffering effect of physical activity was evident among younger participants regardless of change in conditions, whereas among older adults, activity maintenance was only protective for those with a slower accumulation of chronic conditions. These findings show that long-term engagement in physical activity may protect against the potentially disabling effects of increasing numbers of chronic medical conditions, especially among middle-aged adults.

To our knowledge, no studies have explicitly tested the longitudinal interactions of physical activity and chronic conditions on functional limitations in the same sample. However, the present results are consistent with expectations from studies examining both predictors individually. Specifically, Lin and colleagues (29) found among individuals aged 20 years and above that chronic conditions significantly contributed to increases in disability, including mobility and functional limitations, suggesting the deleterious effect of chronic conditions can be observed across adulthood. The present inverse association between long-term physical activity maintenance and functional limitations is also consistent with prior evidence demonstrating protective effects of physical activity on longitudinal increases in limitations (16). This study extends these findings by considering the interactive effects of activity and conditions on functional limitation trajectories. Collectively, these findings suggest that public health efforts to reduce disability burden should target middle-aged as well as older adults.

The buffering effect of physical activity on the conditions-limitations relationship was conditional on baseline age. Specifically, protective effects in those with relatively faster accumulation of chronic conditions diminished with age. These findings do not preclude physical activity benefits among older adults—physical activity interventions are shown to be effective in reducing severe mobility disability in older adults (cf (30).). Instead, these findings suggest that accounting for slower or faster accumulation of chronic conditions can help discriminate between older adults who would likely benefit most from physical activity and those who may require additional (or alternative) intervention strategies to minimize disability, respectively. Moreover, as the protective effect of physical activity maintenance becomes increasingly contingent on a slower rate of accumulation of chronic conditions with advancing age, targeting activity maintenance among midlife adults with single or multiple chronic conditions represents a viable intervention strategy to forestall late-life disability. Other protective factors, such as a rich social network, could also be leveraged to increase effectiveness (31).

There are some limitations to this study. First, chronic conditions were assessed using a basic count variable. Several studies have emphasized the differential impact of specific disease combinations on physical activity participation and disability outcomes (7,19,32). Thus, although the current results show that changes in numbers of conditions alone predict changes in functional limitations, further exploration of disease clusters and their potential impact on disability trajectories is warranted. Second, this study did not account for variability in trajectories of the predictors and outcome. For example, prior work has demonstrated that there are multiple, distinct patterns of change in both activity and mobility over time (33,34). This study examined linear trends as a first step toward disentangling the between- and within-person factors that contribute to the change in functional limitations. Future studies investigating the interactions of various patterns of activity, chronic conditions, and disability will be critical for better understanding their dynamics. Further, as all study variables were collected simultaneously, the direction of the observed interactions cannot be definitively stated.

Third, older adults with slower declines in activity over time are more likely to have maintained activity earlier in midlife (35). Hence, the possibility that the observed protective effect of activity maintenance among older adults arose from a minimization of the change in conditions during midlife cannot be ruled out. It is also possible that the type of conditions that tend to accumulate later in life may be more debilitating than those earlier; however, the fact that both younger and older participants had similar increases in limitations as a function of increasing conditions (depending on physical activity behavior) suggests that it is the rate of change, rather than the conditions per se, that may account for the increase in functional limitations over time. Finally, the participants in this study were predominantly White; these results, therefore, may not generalize to Minority populations.

In sum, this study examined the moderating role of long-term engagement in physical activity in the longitudinal relationship between chronic medical conditions and functional limitations in a large, age-diverse sample of adults. Overall, the findings suggest that long-term maintenance of physical activity may protect against age-related increases in functional limitations over time, even in the context of increasing numbers of chronic conditions. The age dependence of this buffering effect is particularly informative for older adults, among whom it appears to weaken with steeper increases in chronic conditions. Thus, promoting long-term physical activity participation in midlife and beyond may be a viable approach to reducing the functional impact of chronic conditions and the progression of functional limitations in older adults, which may ultimately improve overall quality and quantity of life.

## Funding

This work was supported by a grant to E.M.F. (R01-AG041750) from the National Institute on Aging. K.M. was supported by a grant from the National Institute on Drug Abuse (K01DA039288). The MIDUS 1 study (Midlife in the United States) was supported by the John D. and Catherine T. MacArthur Foundation Research Network on Successful Midlife Development. The MIDUS 2 and MIDUS 3 research was supported by grants from the National Institute on Aging (P01AG020166, R37AG027343). The research was further supported by the following grants: M01-RR023942 (Georgetown) and M01-RR00865 (UCLA) from the General Clinical Research Centers Program and UL1TR000427 (UW) from the National Center for Advancing Translational Sciences (NCATS), National Institutes of Health.

## Conflict of interest statement

None declared.
